# Floral Volatiles from *Vigna unguiculata* Are Olfactory and Gustatory Stimulants for Oviposition by the Bean Pod Borer Moth *Maruca vitrata*

**DOI:** 10.3390/insects8020060

**Published:** 2017-06-09

**Authors:** Bo Feng, Kai Qian, Yong-Jun Du

**Affiliations:** 1 Institute of Health and Environmental Ecology, Wenzhou Medical University, University Town, Wenzhou 325035, Zhejiang, China; fb@wmu.edu.cn (B.F.); qk@wmu.edu.cn (K.Q.); 2 Institute of Pesticide and Environmental Toxicology, Zhejiang University, 866 Yuhangtang Rd., Hangzhou 310058, Zhejiang, China

**Keywords:** floral odor, β-caryophyllene, *Maruca vitrata*, oviposition, *Vigna unguiculata*

## Abstract

We investigated the role of floral odors from cowpea, *Vigna unguiculata* (L.), in mediating oviposition of the bean pod borer moth, *Maruca vitrata*, a serious pest of grain legumes that flies to host plants at the flowering stage and oviposits onto flowers and buds. The flower of the host plant *V. unguiculata* was a stimulus for egg-laying by *M. vitrata* in an oviposition bioassay. Commercial longifolene, β-caryophyllene, linalool, geraniol, and (*Z*)-3-hexenyl acetate were used as stimulus. Each one elicited dose-dependent electroantennogram responses in female *M. vitrata*, and all but longifolene stimulated oviposition, when presented singly. Beta-caryophyllene was the most active stimulant, similar to that of the flower of *V. unguiculata*, and eliciting a dose-dependent oviposition response. Either olfaction or gustation was sufficient to mediate an oviposition response to *V. unguiculata* floral volatiles: intact *M. vitrata* responded to β-caryophyllene whether or not they could contact the source of the volatiles, and females with amputated antennae responded if allowed to contact the source. We believe this is the first demonstration in a moth where β-caryophyllene from the host plant was able to mediate an oviposition response. As β-caryophyllene is widely expressed by non-host plants, we suggest that its role in stimulating oviposition could be exploited as part of a push–pull strategy for pest management in which β-caryophyllene-expressing non-host plants provide a population sink for *M. vitrata*.

## 1. Introduction

Phytophagous Lepidoptera use a hierarchy of semiochemical cues to locate host plants and to determine their suitability for oviposition, followed with a sequence of behavior that involves searching, orientation, encountering, landing, surface evaluation, and acceptance [1,2,3]. Much research has been focused on the role of antennal olfactory receptors for plant volatiles, including floral odors, which enables orientation and movement towards the host plant from a distance [4,5]. By contrast, after an insect alights on a plant, contact perception of its physical and chemical characteristics will become paramount in determining the plant’s suitability for oviposition. Fewer volatile secondary plant metabolites are recognized by contact chemoreceptors, usually on the insect’s ovipositor, tarsi, or mouth parts [6]. Host plant compounds that stimulate oviposition have been found in several moth species [7,8,9], and a range of chemicals such as pyrrolizidine alkaloids [10] and (−)-germacrene D [11] have been identified as stimulants.

The bean pod borer, *Maruca vitrata* (Fabricius) (Lepidoptera, Crambidae), is oligophagous on legumes in the tropics and subtropics, and is a serious pest of grain legumes [12,13]. Newly emerged adult *M. vitrata* fly to host plants during the flowering or budding stage. Females usually oviposit onto flower buds or flowers [14], allowing the larvae to cause severe feeding damage to flower buds, flowers, and developing bean pods [15]. *M. vitrata* often distributes eggs singly on the flowers of the cowpea, *Vigna unguiculata* (L.) Walp. The flowers provide abundant nutrition to the adult, which in turn contributes to ovary and egg development [16]. In West Africa, *M. vitrata* appears to migrate from the south to the north over the course of several months, a movement most likely explained by the phenology of flowering of leguminous trees [17].

Floral scent often plays a key role in acting as a selective attractant to plants, particularly where floral morphology itself does not impose selection on flower visitors [18]. Male and female moths are attracted to flowers as food sources. The importance of floral odor cues in this behavior is well documented among noctuid pest species [19,20]. In addition, some studies reported the effect of flower scent on the oviposition of moths, such as *Helicoverpa armigera* [21,22] and *Heliothis virescens* [23]. Given the close association of the life history of *M. vitrata* with the flowering of its host plants, floral volatiles could play an important role in host location and selection, as has been found in the pollen beetle, *Meligethes aeneus* (Fab.), a temperate pest of the buds and flowers of oilseed rape *Brassica napus* L. [24]. Evidence from laboratory experiments show that the larvae of *M. vitrata* are attracted more to host flowers as opposed to pods [25,26]. Indeed, as far as we are aware, research on the effect of flower scent on oviposition in Lepidoptera is rare and lacking. Studies have been focused more on the role of less volatile secondary metabolites of host and non-host plants detected by contact chemoreception [27,28]. The volatiles emanating from host crops of *M. vitrata* have been studied, and they play a key role in the mating behavior of *M. vitrata* possibly by augmenting sex responses in both sexes [29]. However, the effect of floral odors on oviposition behavior in *M. vitrata* remains unknown. Indeed, as far as we are aware, research on the effect of flower scent on initiating moth’s oviposition in Lepidoptera is rare and lacking.

The number of compounds in the volatiles of cowpea flower was about 109 and they were classified into six categories on the basis of biosynthetic origin: aliphatics, aromatics, monoterpenes, sesquiterpenes, norisoprenoids, and other miscellaneous compounds that were present, but did not share any striking similarities [30]. Of these volatiles linalool, β-caryophyllene and geraniol are widespread compounds of floral scents and have been reported in flower of many plant taxa, including many legume species [31]. These three volatiles were found to be important factors in the pollination of flowers by bees [32,33,34], flower thrips [35], garden chafer [36], and hummingbirds [37]. In the present study, specific floral volatiles known from literature were used to investigate the role of cowpea floral odor in mediating oviposition by *M. vitrata*. Longifolene is a known floral volatile in Amaryllidaceae, Araceae, Musaceae, and Rosaceae [31] and has been found in flowers of *V. unguiculata* in our extracts. (*Z*)-3-Hexenyl acetate is one of the most abundant compounds in the bean leaf (Lwande et al. 1989) and was also reported in cowpea floral volatiles [30]. Both longifolene and (Z)-3-hexenyl acetate were used in our study to determine if they could mediate oviposition by *M. vitrata.*

The specific objectives of the work described here were (1) to investigate the role of floral odor from the cowpea, *V. unguiculata* in mediating oviposition of *M. vitrata*; (2) to evaluate the compounds in the floral odor responsible for oviposition; (3) to assess the roles of olfaction and gustation in sensing these compounds; (4) to underpin the future development of semiochemically based technologies for control of this pest.

## 2. Methods and Materials

### 2.1. Insects

Cowpea pods with *Maruca vitrata* larvae were collected from cowpea plants from the fields in Ouhai, Wenzhou, China, from June to October in 2009–2011, and they were placed in plastic cups with a 250 mL volume. Plastic cup was covered with a square gauze (15 cm × 15 cm) and placed in a climate chamber (8 m × 3 m × 3 m) at 25 ± 1 °C, 75 ± 5% relative humidity, and 14:10 h (L:D) photoperiod. Plastic cups were checked daily to monitor the emergence of *M. vitrata* moths. Male and female moths were separated immediately after eclosion according to the morphology of the abdominal tip [38]. Adults were kept in screen cages (30 cm × 25 cm × 25 cm) and fed a 10% glucose aqueous solution that was replaced daily.

### 2.2. Plants

From June 2009 to August 2011, *V. unguiculata* was sown in an experimental field (4 m × 4 m) near the Chashan campus of Wenzhou Medical University. Seeds were planted in groups of three, with each group being separated by a length of 30 cm. When the plants grew to a height of about 50 cm, those infested with pests were removed. A wooden frame (4 m × 4 m × 2 m) covered with white gauze (size of mesh about 1 mm) was set up around the plants to prevent pest infestation. The use of pesticides and other agrochemicals was prohibited to avoid contaminating and altering the volatiles taken off from the plants. To provide nutrients for the plant growth, standard organic vegetable food was used before sowing, at a plant height of about 30 cm, and after flowering.

### 2.3. Commercial Chemicals Used as Stimuli

Linalool, geraniol, longifolene, and (*Z*)-3-hexenyl acetate were purchased from Sigma-Aldrich (St Louis, MO, USA), α-caryophyllene and β-caryophyllene from Tokyo Chemical Industry Co., Ltd. (Tokyo, Japan), and paraffin oil from Tianjin Kemiou Chemical Reagent Co., Ltd. (Tianjin, China). The purity of all chemicals was greater than 97%. Linalool, geraniol, longifolene, (*Z*)-3-hexenyl acetate, and β-caryophyllene were diluted with paraffin oil to concentrations 10^−1^, 10^−2^, 10^−3^, 10^−4^, and 10^−5^ (V/V). A synthetic blend was made from equal parts of those five diluted chemicals (10^−1^ concentration).

### 2.4. Electroantennogram (EAG) Recording

Recordings of whole-antenna electrical activity in response to volatile stimuli were made according to the standard technique described by Yan [39]. Antennae for EAG recordings were amputated from the head at their base. After removing the terminal segment, antennae were attached to each side of a two-pronged electrode “fork” recording probe (base of antenna at the ground position) using a small amount of electrode gel. The recording probe was connected to the pre-amplifier of a Syntech EAG recording system. From there, signals were transmitted to an oscilloscope for monitoring and through a Syntech signal acquisition controller, IDAC-2, to a computer for recording and analysis using Syntech EAG 2000 software. Stimuli were presented by introducing odoriferous air into a continuous air stream (1200 mL/min) cleaned by activated charcoal, humidified by passage through water, and directed at the antenna through a Pasteur pipette. Odors were introduced in air passed through a glass dropper containing a 30 mm × 4 mm strip of filter paper impregnated with 10 µL of a solution of the test compound in paraffin oil. For each stimulus, a 0.1 s air puff of odoriferous air (air flow 40 mL/min) was delivered into a hole in the Pasteur pipette carrying the clean air stream. To avoid sensory adaptation, there was at least a 30 s lapse between two stimuli. Five concentrations (10^−1^, 10^−2^, 10^−3^, 10^−4^, and 10^−5^) of linalool, geraniol, longifolene, (*Z*)-3-hexenyl acetate, and β-caryophyllene were used in the experiment. Antennae from six mated female moths were tested for every concentration of each chemical. Paraffin oil was used as control, and its EAG response was subtracted from the test compound. As the response of antennae declined during the course of the experiment, a 1% concentration (V/V) of (*Z*)-3-hexenyl acetate was used as a reference. Responses of antennae to all tested chemicals were standardized with response to the reference.

### 2.5. Dissection of Reproductive System

One- to three-day-old female moths were anaesthetized with CO_2_, and the abdomens were removed. The reproductive system was dissected in PBS solution under a microscope. Photos were taken with Nikon SMZ1500.

### 2.6. Moth Copulation

Eight pairs of male and female moths of the same age were introduced into the screen cage and allowed to freely move around and mate. Copulation was monitored every night (19:00–06:00) using a digital handy camera (DCR-TRV27, SONY, Tokyo, Japan) with a super night shot. Mating pairs were transferred to a cylindrical plastic cup. Three- to five-day-old mated females were used for the oviposition bioassay in the following morning. Five replicates were conducted.

### 2.7. Effect of Host Plant Flower on Oviposition

A mated female was transferred to a plastic cup with a 250 mL volume, which was covered with a piece of gauze (15 cm × 15 cm) to prevent the female moth escaping. Two fresh-cut healthy flowers of *V. unguiculata* with the stems in a small (10 mL) cup with fresh water inside the big cup were used as the oviposition stimulus. A plastic cup without any flower (blank group) was used as a control. Each moth was fed with a 10% glucose solution that was maintained with absorbent cotton. Eggs laid in the 250 mL cup, the 10 mL cup, the absorbent cotton, and the gauze were counted daily under a stereomicroscope. The glucose solution, flowers of *V. unguiculata*, and plastic cups were replaced daily (17:00 h) until the moth died. Ten mated moths were used for each treatment as ten replicates.

### 2.8. Effect of Components of *V. unguiculata* Floral Volatiles on Oviposition

To test the effect of components of *V. unguiculata* floral volatiles on oviposition, 20 µL of diluted linalool, geraniol, (*Z*)-3-hexenyl acetate, longifolene, and β-caryophyllene (10^−1^ concentration) with paraffin oil, or their synthetic blend with the same volume was placed on a filter paper (30 mm × 10 mm), which was hung as the oviposition stimulus. Paraffin oil (20 µL) placed on the double-folded filter paper was used for control. A mated female was maintained in a 250 mL plastic cup and fed with 10% glucose solution as before. Eggs laid in the cup, on the filter paper, on the absorbent cotton, and on the gauze were counted twice a day (06:00 h and 17:00 h), and food, stimulus, and plastic cup were replaced daily (17:00 h) until the moth died. In addition, α-caryophyllene and β-caryophyllene (10^−1^ concentrations) were used as stimulus, but eggs were counted daily. Ten mated moths were used for each treatment as ten replicates.

### 2.9. Effect of Concentrations of β-Caryophyllene on Oviposition

To test the effect of concentrations of β-caryophyllene on oviposition, 20 µL portions with different concentrations of β-caryophyllene (10^−1^, 10^−3^, and 10^−5^ concentrations) diluted with paraffin oil was placed on a filter paper (30 mm × 10mm), and the double-folded filter paper was hung as the oviposition stimulus. Paraffin oil (20 µL) placed on the double-folded filter paper was used for control. Either a mated or unmated female was maintained in a plastic cup and fed with 10% glucose solution as before. All eggs were counted, and the food, stimulus, and plastic cup were replaced daily (17:00 h) until the moth died. Ten mated moths were used for each treatment as ten replicates.

### 2.10. Testing the Roles of Olfaction and Gustation in the Reception of β-Caryophyllene

The filter paper (30 mm × 10 mm) with 20 µL of β-caryophyllene with a 10^−1^ concentration was either hung directly in the oviposition cup as before (‘with contact’ treatment) or placed into a 2 mL Eppendorf tube, which was punctured with 100 holes each 0.50 mm in diameter was hung in the oviposition cup (‘without contact’ treatment). For the ‘non-antennae’ treatment, the antennae were cut at the base with scissors under stereomicroscope, while the female moth was anaesthetized on ice, and for the ‘antennae’ treatment, the antennae were left as is on the moth. There were 4 combinations for the stimulus and mated female: contact × non-antennae, non-contact × non-antennae, non-contact × antennae, and contact × antennae. Paraffin oil × antennae was used as control. The anaesthetized and mated female (with or without antennae) was maintained in a plastic cup and fed with 10% glucose solution as before. Eggs laid in the 250 mL cup, and on the filter paper (the Eppendorf tube), the absorbent cotton, and the gauze, were counted daily, and the food, stimulus, and plastic cup were replaced daily (17:00 h) until the moth died. Ten mated moths were used for each treatment as ten replicates.

### 2.11. Data Analysis

Statistical analysis was conducted using SPSS17.0. One-way analysis was used to determine the variance for raw data with three or more treatments. If variances were homogeneous, then multiple comparisons using Duncan’ test was used. If variances were non-homogeneous, then Tamhane’s T2 test was performed to analyze significant differences between the data. Data from two treatments were analyzed by a Student’s *t*-test. The critical *p*-value for each test was set at 0.05.

## 3. Results

### 3.1. Egg Maturation and Female Mating Frequency by Age

**Figure 1:**
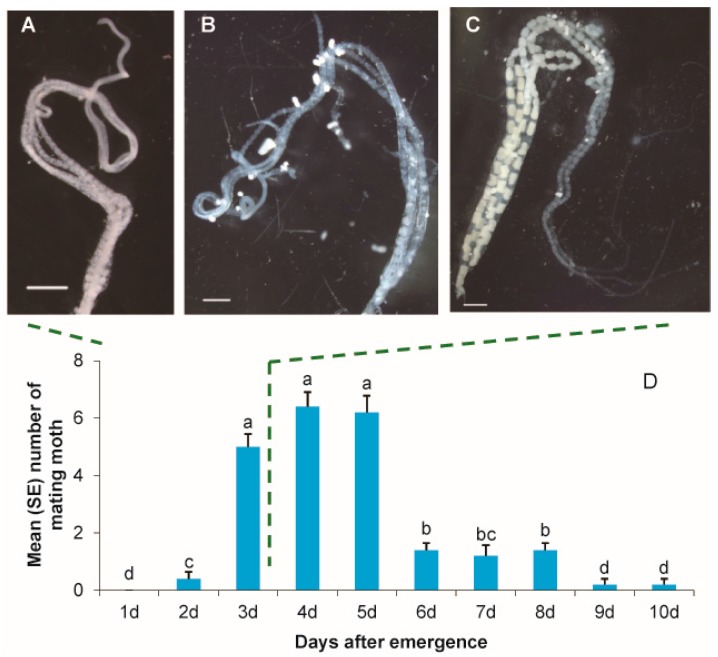
Egg maturation and female mating frequency by age. (**A**–**C**) The ovary of 1-, 2-, and 3-day-old females; scale in lower left is equal to 2.0 mm. After feeding, the ovary gradually developed in the body, and there were some mature eggs in ovaries of 3-day-old females. (**D**) The mean number of moth pairs observed copulating each day (means with the same latter are not significantly different; *p* > 0.05, one-way ANOVA with Duncan’ test). Error bars signify SE. Nearly all moths copulated at the age of 3 days or older.

The ovary of *M. vitrata* was long and eggs in the ovary were nearly all transparent at adult emergence (Figure 1A). After feeding, yolk formation was accelerated and eggs became opaque because of the light scattering from the yolk (Figure 1B). Some mature eggs were found in three-day-old females (Figure 1C). Although a few moths were mating at two days old, the copulation of most *M. vitrata* started on Day 3 or later (Figure 1D), at which times the ovaries of mated females were mature.

### 3.2. Oviposition by Age with and without Flowers Present

**Figure 2:**
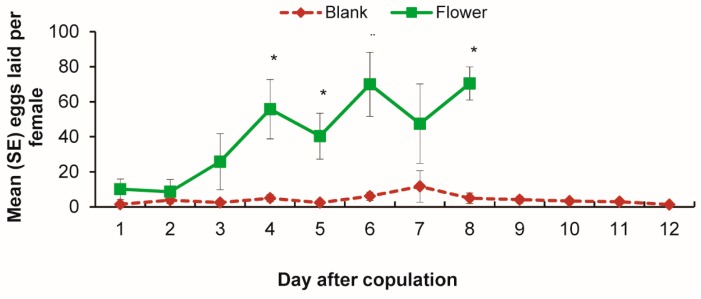
Oviposition by age with and without flowers present. Error bars signify SE. Significance of difference between blank and flower treatment indicated by “*”, *p* < 0.05, Student’s *t-*test. From the third day after copulation, the number of eggs laid by the moths exposed to host flowers was greater than those in blank group.

The flower of the host plant *V. unguiculata* was the stimulus for egg-laying in the oviposition bioassay (Figure 2). From the third day after copulation, the number of eggs laid by the moths exposed to host flowers was evidently increased. In contrast, females in the control group (blank group) laid a few eggs every day, and the number of eggs was unchanged even 12 days after copulation (Figure 2). Female exposed to host flowers laid significantly more eggs than those in the control (blank) group from the fourth days (*p* ≤ 0.006, except Day 7). The host flower stimulated oviposition by mature females, but ovary and egg development proceeded in the same way for all females whether with or without flowers in the chamber.

### 3.3. EAG Responses of Female Antennae to Components of *V. unguiculata* Floral Volatiles

**Figure 3:**
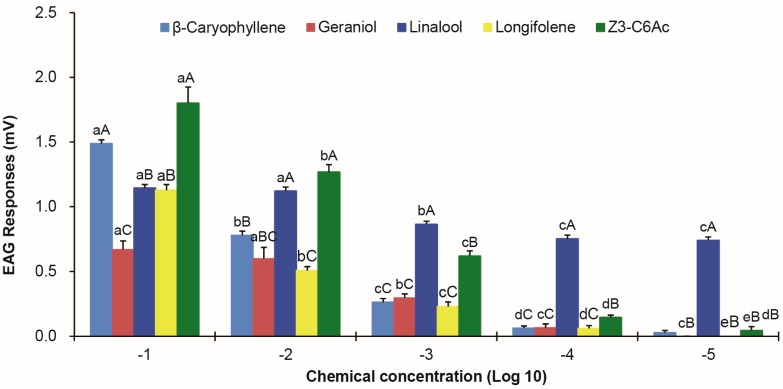
Mean electroantennogram responses recorded from female *M.* *vitrata* antennae elicited by 10 μL of a paraffin oil solution of floral volatiles at decreasing concentrations. Error bars signify SE. Significance among different concentrations of the same volatile and among different volatiles of the same concentration are indicated by lower case and upper case letters, respectively. Means are significantly different if followed by a different letter (*p* < 0.05, one-way ANOVA with Duncan’ test).

Female antennae showed evident EAG responses to commercial preparations of five components identified in the floral volatiles of *V. unguiculata* presented in paraffin oil. Significant decreases were found for the moth response when these chemicals were gradually diluted with paraffin oil (*p* < 0.05; Figure 3). However, decreased rates of moth response were diversified for these chemicals. Significant decreases were found in linalool and geraniol with 10^−3^ and 10^−4^ concentrations, in β-caryophyllene with 10^−2^ to 10^−4^ concentrations, and in longifolene and (*Z*)-3-hexenyl acetate with every diluted concentration (*p* ≤ 0.014). Slopes and regression values of EAG responses as a function of concentration testing for linalool, geraniol, β-caryophyllene, longifolene, and (*Z*)-3-hexenyl acetate were −0.117 (R^2^ = 0.898), −0.187 (R^2^ = 0.954), −0.364 (R^2^ = 0.869), −0.270 (R^2^ = 0.865), and −0.464 (R^2^ = 0.955), respectively. Although the response to linalool was significantly smaller than β-caryophyllene and (*Z*)-3-hexenyl acetate with a 10^−1^ concentration, it was still significantly larger than other chemicals at low concentrations (10^−4^ and 10^−5^) (*p* < 0.001).

### 3.4. V. unguiculata Floral Volatiles as Stimuli on Female Oviposition

**Figure 4:**
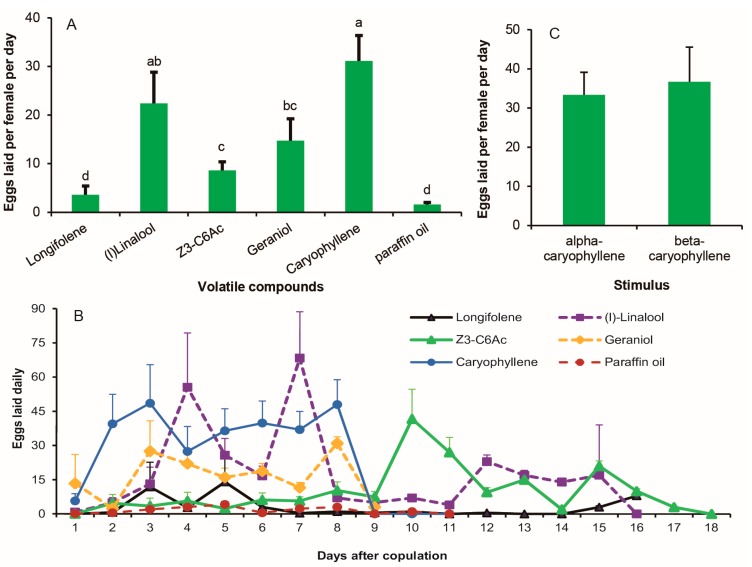
*Vigna unguiculata* floral volatiles as stimuli on female oviposition of *M. vitrata.* (**A**) The number of eggs laid per female per day elicited by 20 μL of a paraffin oil solution of floral volatiles applied to paper. (**B**) The number of eggs laid every day after copulation. Caryophyllene with the strongest stimulation on female oviposition for tested volatiles. (**C**) Oviposition stimulation of isomer of Caryophyllene. Error bars signify SE. Significance among different volatiles are indicated by lower case letters. Different letter means significant difference (*p* < 0.05, one-way ANOVA with Duncan’ test).

Four components of *V. unguiculata* floral volatiles—β-caryophyllene, geraniol, linalool, and (*Z*)-3-hexenyl acetate—significantly stimulated egg-laying of *M. vitrata* when compared to paraffin oil controls (*p* ≤ 0.001; Figure 4A). The egg-laying response to β-caryophyllene was not only the most rapid, but also the strongest. All eggs were laid within the first eight days of the bioassay (Figure 4B). For the other three oviposition-stimulating chemicals, egg-laying responses were not evidently different from each other, with the exception of the (*Z*)-3-hexenyl acetate between the 8th and 11th day. Within these days, egg-laying stimulated by (*Z*)-3-hexenyl acetate was more than that by the geraniol and linalool (Figure 4B). The effect of isomers of caryophyllene on the oviposition was the same (Figure 4C). Longifolene did not stimulate egg-laying of *M. vitrata* compared to the control (Figure 4A).

### 3.5. Stimulation of Host Floral Volatiles on Female Oviposition Restricted in Night

**Figure 5:**
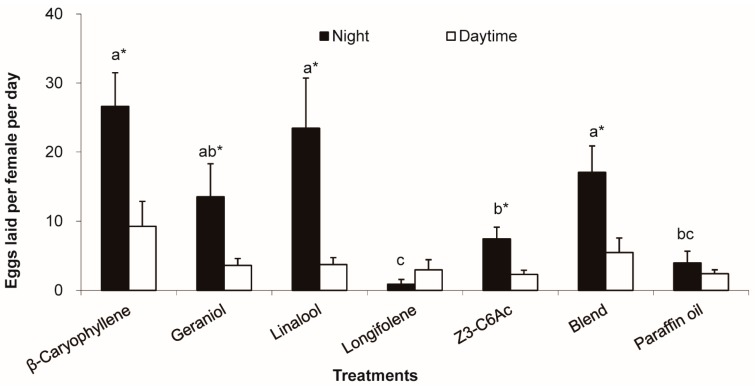
Eggs laid during the day (06:00–17:00 h) and the night (17:00–06:00 h) by female *M. vitrata* elicited by 20 μL of a paraffin oil solution of floral scents applied to paper. Error bars signify SE. Significance among different volatiles and between night and daytime indicated by lower case letters and “*”. Different letter and “*” means significant difference (*p* < 0.05, one-way ANOVA with Duncan’ test for different volatiles and Student’s *t-*test for night and daytime).

Females in control (paraffin oil) group laid very few eggs with no difference between night and day. After being exposed to host floral volatiles (except longifolene), females laid significantly more eggs at night than during the day (*p* ≤ 0.011). The number of eggs laid at night was significantly greater than that of the control (paraffin oil) group, when the mated females were exposed to β-caryophyllene, linalool, and the blend (*p* ≤ 0.023). For example, the number of eggs increased 14 times for β-caryophyllene treatment at night (*p* = 0.005; Figure 5). However, the number of eggs laid during the day was not significantly increased after host floral volatiles treatment (*p* = 0.169).

### 3.6. Stimulation of β-Caryophyllene on Female Oviposition as Strong as Flower

**Figure 6:**
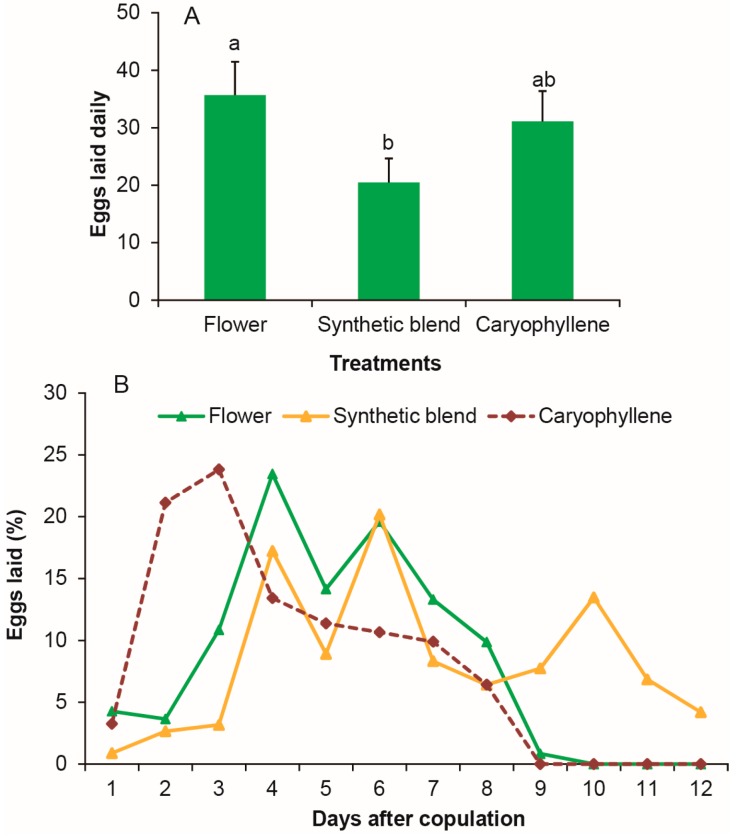
Effect of caryophyllene on oviposition of *M. vitrata* as strong as flower. (**A**) The number of eggs laid per female per day. (**B**) The number of eggs laid every day after copulation. Error bars signify SE. Significance among different volatiles indicated by lower case letters. Different letter means significant difference (*p* < 0.05, one-way ANOVA with Duncan’ test).

Female oviposition from β-caryophyllene was as strong as that from two flowers (*p* = 0.582; Figure 6A). However, the synthetic blend of all five main components of *V. unguiculata* volatiles was significantly less active in the oviposition bioassay than that of the two *V. unguiculata* flowers (*p* = 0.047; Figure 6A). The oviposition stimulation of β-caryophyllene was faster than that of *V. unguiculata* flowers, but the oviposition stimulation of the synthetic blend was delayed and weaker when compared to the stimuli from flowers (Figure 6B).

### 3.7. Dose-Response Relationship for β-Caryophyllene as an Oviposition Stimulus

**Figure 7:**
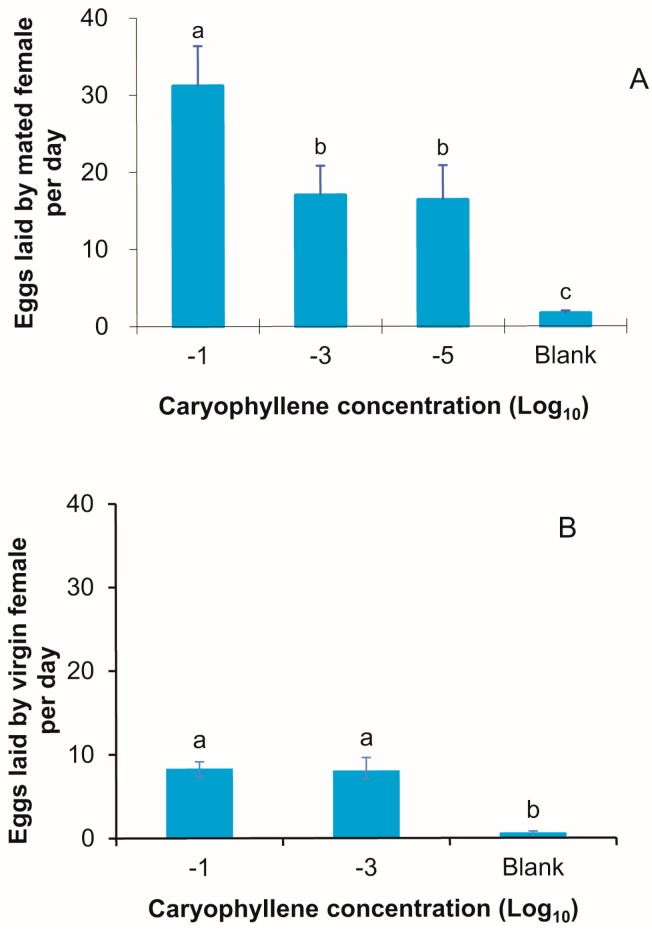
Effect of caryophyllene dosage on oviposition of mated (**A**) and unmated (**B**) *M. vitrata* elicited by 20 μL of a paraffin oil solution of caryophyllene applied to paper. Error bars signify SE. Significance among different volatiles indicated by lower case letters. Different letter means significant difference (*p* < 0.05, one-way ANOVA with Duncan’ test).

The response elicited by β-caryophyllene in the oviposition bioassay was dose-dependent, in agreement with the EAG results. Significantly more eggs were laid per day in the presence of β-caryophyllene with a 10 ^−1^ concentration compared with that with 10^−3^ or 10^−5^ concentrations (*p* ≤ 0.028), but the number of eggs laid in a 10^−5^ concentration was significantly greater than that in the control group (paraffin oil) (Figure 7A, *p* < 0.001). Interestingly, unmated females laid some eggs in response to β-caryophyllene (Figure 7B, *p* < 0.001).

### 3.8. Roles of Olfaction and Gustation in the Mediation of the Floral Volatile Oviposition Stimulus

**Figure 8:**
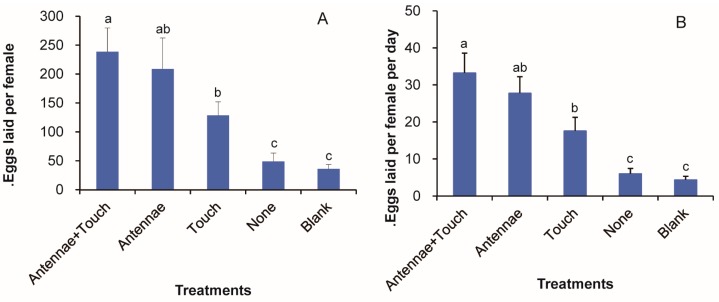
Testing the effect of antennal ablation and access to the odor source on oviposition of *M. vitrata* elicited by 20 μL of a 10% paraffin oil solution of caryophyllene applied to paper. Antennae + Touch = female with intact antennae and with access to treated filter paper; Antennae = intact females responding to treated filter paper confined in ventilated tube; Touch = females lacking antennae with access to treated filter paper; None = Females without antennae responding to treated filter paper confined in ventilated tube; Blank = filter paper with paraffin oil. Error bars signify SE. Significance among different volatiles indicated by lower case letters. Different letter means significant difference (*p* < 0.05, one-way ANOVA with Duncan’ test).

After being stimulated by β-caryophyllene with a 10^−1^ concentration, intact females with access to treated filter paper laid about the same number of eggs as intact females without access to treated filter paper (without contact, *p* = 0.528), but laid significantly more eggs than females lacking antennae and with access to treated filter paper (*p* = 0.04) (Figure 8). When denied access to treated filter paper, moths without antennae laid significantly fewer eggs than moths with antennae or/and with access to treated filter paper (*p* ≤ 0.01). In addition, females without antennae and access to treated filter paper laid no more eggs than intact females stimulated with paraffin oil (the control group, *p* = 0.314) (Figure 8).

## 4. Discussion

Insect egg deposition is the start of the establishment of a new herbivore generation. Newly hatched larvae of many insects are relatively immobile and depend on the judicious behavior of their mother to find the best source of food for their successful growth and development. Female moths typically lay eggs on the larva host. The larvae of *M. vitrata* feed on the flower buds, flowers, and pods of *V. unguiculata* [13,40,41]. The incidence of *M. vitrata* larvae increases with the initiation of flowering. Maximum *M. vitrata* population is observed during flowering, and most 1st and 2nd instar larvae are observed on flowers [42,43,44]. In our bioassay, whole flowers of *V. unguiculata* strongly stimulated oviposition of the bean pod borer.

Linalool, β-caryophyllene, geraniol, and Z3-C6:Ac are common floral volatiles and have been found in many genera of family Fabaceae, such as genera *Lathyrus*, *Lupinus*, *Medicago*, *Robinia*, and *Trifolium* [45,46,47,48]. Each of these compounds elicited dose-dependent electrophysiological responses in the antennae of female *M. vitrata*. Although longifolene was not found in flowers of the family in former studies, it was found in our experiments. The antennae of female *M. vitrata* responded to longifolene in the electrophysiological test.

Several studies have shown that the production of β-caryophyllene, a sesquiterpene increased through herbivore feeding in cotton [49] and bean [50], and by egg deposition in leguminous plants [51] and elm [52] to attract parasitoids of herbivore. Some predators, such as the harlequin ladybird also use it as a cue to locate prey and oviposition sites [53,54]. Beta-caryophyllene was also the attractant for some herbivorous insects such as the weevil [55] and the damson hop aphid [56]. In our study, β-caryophyllene was the most active oviposition stimulant of *M. vitrata* among the tested floral volatiles. Beta-caryophyllene alone stimulated oviposition as strongly as the intact flowers. Its oviposition stimulation was faster than flowers, and it elicited a dose-dependent oviposition response. The oviposition stimulation of β-caryophyllene was also found in *Cameraria ohridella*, where the females’ oviposition increased through the chemicals released from the blossoms of the host tree [57]. In fact, oviposition behavior influenced by one or several common plant volatile compounds has been found in many insects, such as the Mediterranean fruit fly *Ceratitis capitata* [58,59], and the moths *Helicoverpa armigera*, *Spodoptera litura*, and *Chilo partellus* [60,61]. For example, common plant volatile compounds (Z)-3-hexenyl acetate, (3E)-4,8-dimethyl-1,3,7-nonatriene, and linalool were found to significantly enhance female oviposition of *Chrysopa phyllochroma* [62]. *Manduca sexta* females oviposited more on plants emitting (−)-linalool (alone or in mixtures) than control plants, while plants emitting (+)-linalool (alone or in mixtures) were less preferred than control plants [63]. By contrast with β-caryophyllene, the sesquiterpene longifolene was not significantly active in the oviposition bioassay when presented singly. The antennal response of female *M. vitrata* to longifolene in the electrophysiological test might encode specificity, as it permits females to discriminate between the non-host plant and host plant. (*Z*)-3-Hexenyl acetate is one of the most abundant volatiles released by *V. unguiculata*, comprising 28.8% of volatiles collected from 3-week-old cowpea plants [64] and 40% of the volatiles emitted by their leaves, a proportion that increases rapidly if the leaves are injured [65]. The release of (*Z*)-3-hexenyl acetate by *V. unguiculata* leaves is consistent with their activity in our oviposition bioassay.

*M. vitrata* is a nocturnal moth and its behavior, including feeding and mating, has always been found during nighttime [66]. In our study, after being exposed to host floral volatiles, females laid significantly more eggs at night, and the number of eggs increased 14 times for β-caryophyllene treatment. The stimulation of oviposition during the night was in accordance to the nocturnal habitats of *M. vitrata* moth*.* In nature, blossoms of *V. unguiculata* are found between the hours of 6:00–12:00 a.m. [67]. The blossom of *V. unguiculata* was seemingly inconsistent with the oviposition time of *M. vitrata*. In fact, the blooming of *V. unguiculata* flower was gradually accomplished from 9:00 p.m. in our observation. In the process of *V. unguiculata* flower blooming, floral volatiles might be found by nocturnal moth, including *M. vitrata*. Female *M. vitrata* laid eggs during the day. The emergence of *M. vitrata* moth was also found during the day but the number of moths that emerged during this period was only about 20% [66]. Diurnal behaviors were also found in other nocturnal moths. Mating and oviposition during the day were found in *Plutella xylostella* in the winter [68]. Throughout the day, *Lymantria dispar* virgin females exhibit calling behavior to attract males [69]. The number of eggs laid by female *M. vitrata* during the day was not significantly increased with host floral volatile treatment. The exact mechanism of this phenomenon is unknown, but it was able to shut down the recognition of moth to the floral volatiles during the day. A diurnal rhythm for expression of olfactory genes had been found in many nocturnal moths. For example, gene expression of *OR11*, *OR18*, *Orco*, *ABP2*, *OBP1,* and *PBP1* increased after light-off and peaked after four hours in *Spodoptera exigua* [70]. Beta-caryophyllene, linalool, geraniol, and (*Z*)-3-hexenyl acetate had oviposition stimulant activity for *M. vitrata* moths when they were active on their own, but all volatiles were common among non-hosts [31]. Oviposition stimulants might not be host-specific if host-specificity is determined by other cues during host location flights; rather, they should indicate the quality of the host plant [71] and allow oviposition to be synchronized with plants at the appropriate developmental stage. The decreased level of volatiles could have contributed to the low quality of the host plant and decreased the number of eggs that the female laid. Other moths were also responsive to multiple volatiles as oviposition stimulants [72,73]. Although the number of eggs under 10^−1^ β-caryophyllene stimulus was the same as that of the flower in our study, we cannot conclude that β-caryophyllene alone has the same stimulation as does an entire flower in nature, as the active concentration of β-caryophyllene is narrow. When the concentration of β-caryophyllene was decreased, the number of eggs was dramatically decreased and was much lower than the number elicited by the flower, suggesting that additional volatiles from the host plant may be active.

There were examples where synergy occurred in the response to multiple volatiles as in *Heliothis armigera* [22], and where it did not occur, as in *Cochylis hospes* [27]. The presence of synergy in response to multiple volatiles was likely to indicate the unreliability of single volatile cues as the indicators of the plant quality required by the insect for rearing the next generation. In *M. vitrata*, there appeared to be an absence of synergy between oviposition stimulants with the same volume, which might have resulted from the synthetic blends following the unnatural composition. The synergy between oviposition stimulants might be confirmed by tests with volatiles in natural proportions. The use of more than one volatile as an oviposition cue was likely to be an advantage even for an oligophagous insect, as different hosts might release different volatiles in different qualities.

Intact *M. vitrata* responded to oviposition stimuli whether or not they could contact the source of the volatiles. We believe this is the first demonstration in a moth where oviposition behavior was able to be initiated only by host-plant floral volatiles sensed by olfaction. Olfactory detection of plant volatiles in *M. vitrata* was most likely associated with the sensilla of the distal flagellar segments of the antenna, which is important in mediating the oviposition stimulus provided by the host plant [74]. Few plant volatile secondary metabolites have been recognized by gustatory receptors on the insect’s ovipositor, tarsi, or mouth parts [1,6]. In our studies, female *M. vitrata* were able to sense the volatile without antennae. There is a mediating role for receptors not on the antennae, and these non-antennal receptors for the oviposition stimulus are gustatory. Gustatory receptors for oviposition stimulants may be located on the tarsi where, in the swallowtail butterfly *Atrophaneura alcinous*, the binding proteins involved in the recognition of oviposition stimulants had recently been characterized [75]. In recent findings, the wing gustatory hair and tracheal architectures of *Drosophila* had been found capable of trapping volatile molecules from the environment [76]. We found evidence that either olfaction or gustation was sufficient to mediate an oviposition response to *V. unguiculata* floral volatiles. The sufficiency of olfaction in mediating the oviposition stimulus for *M. vitrata* contrasted with *P. xylostella* which, when given olfactory but not gustatory stimuli, did not oviposit, but when given gustatory stimuli laid similar numbers of eggs as when given both olfactory and gustatory cues [77]. The use of both olfactory and gustatory cues for oviposition perhaps in part explains why not all eggs were laid on flowers, which is the preferred location for the first and second instar larvae [43], but were scattered on the leaves, flowers, stems, and pods [40]. This oviposition behavior could be attributed to adaptation. If floral oviposition cues are perceived over a longer range by natural enemies, and newly eclosed larvae are capable of moving to the flowers, then females scattering eggs more widely on the plant might reduce overall predation pressure on their progeny.

We showed that certain floral volatiles alone could elicit the oviposition of *M. vitrata*, which might open doors for future research into semiochemical-based control methods for this widespread pest [24]. The effectiveness of a single component, β-caryophyllene, which is widely expressed by non-host plants, could be exploited as part of a push–pull strategy in which oviposition onto β-caryophyllene-expressing non-host plants provides a sink for the *M. vitrata* population. Alternatively, it might be possible to block the olfaction of the volatiles through artificial application of inhibitory compounds, or to alter the phenology of volatile release by the host plant so that moth oviposition lose its synchrony with flowering.

## 5. Conclusions

The bean pod borer, *Maruca vitrata* (Fabricius) (Lepidoptera, Crambidae), is a serious pest of grain legumes in the tropics and subtropics. The larva is a borer in flower buds, flowers, and developing bean pods, making it difficult to manage the pest with chemical insecticides. In our study, β-caryophyllene, a common volatile of plant flowers was found as the most active stimulus in the oviposition of *M. vitrata* among the five compounds tested, as effective as the flower of *V. unguiculata*. Either olfaction or gustation was sufficient to mediate the oviposition response to β-caryophyllene. Our results shed some light on the mechanism of insect oviposition and might open doors for future research into semiochemically based control methods for this widespread pest.
